# Beneficial bacteria inhibit cachexia

**DOI:** 10.18632/oncotarget.7730

**Published:** 2016-02-25

**Authors:** Bernard J. Varian, Sravya Goureshetti, Theofilos Poutahidis, Jessica R. Lakritz, Tatiana Levkovich, Caitlin Kwok, Konstantinos Teliousis, Yassin M. Ibrahim, Sheyla Mirabal, Susan E. Erdman

**Affiliations:** ^1^ Division of Comparative Medicine, Massachusetts Institute of Technology, Cambridge, MA, USA; ^2^ Laboratory of Pathology, Faculty of Veterinary Medicine, Aristotle University of Thessaloniki, Thessaloniki, Greece

**Keywords:** cachexia, sarcopenia, probiotic, microbe, inflammation

## Abstract

Muscle wasting, known as cachexia, is a debilitating condition associated with chronic inflammation such as during cancer. Beneficial microbes have been shown to optimize systemic inflammatory tone during good health; however, interactions between microbes and host immunity in the context of cachexia are incompletely understood. Here we use mouse models to test roles for bacteria in muscle wasting syndromes. We find that feeding of a human commensal microbe, *Lactobacillus reuteri,* to mice is sufficient to lower systemic indices of inflammation and inhibit cachexia. Further, the microbial muscle-building phenomenon extends to normal aging as wild type animals exhibited increased growth hormone levels and up-regulation of transcription factor Forkhead Box N1 [FoxN1] associated with thymus gland retention and longevity. Interestingly, mice with a defective FoxN1 gene (athymic nude) fail to inhibit sarcopenia after *L. reuteri* therapy, indicating a FoxN1-mediated mechanism. In conclusion, symbiotic bacteria may serve to stimulate FoxN1 and thymic functions that regulate inflammation, offering possible alternatives for cachexia prevention and novel insights into roles for microbiota in mammalian ontogeny and phylogeny.

## INTRODUCTION

Cachexia is a wasting syndrome characterized by adipose tissue and muscle atrophy [1-3]. Cachexia is seen in patients with cancer [1], chronic obstructive lung disease [COPD] [4], and multiple sclerosis [MS] causing serious disabilities and premature deaths [5]. Cachexia has been associated with chronic inflammation, in particular involving neutrophils, such as in patients with COPD [4]. Mouse models, such as the *Apc^Min/+^* [ApcMIN] mutant mouse of intestinal polyposis [6], have been utilized to study cancer-associated cachexia due to uncontrolled levels of Interleukin (IL)-6 and other host inflammatory responses [7-10]. Contributions of the gut microbiome to intestinal polyposis have been examined in the ApcMIN mouse model [11-13]. In addition to numerous pre-malignant intestinal polyps and cachexia, ApcMIN mice also display premature thymic involution [14] linked with their early demise at a very young age.

Muscle wasting termed sarcopenia is also a feature of a natural aging process contributing to disability and death [15, 16]. Senility-associated skeletal muscle functional impairment and atrophy have been documented in mice [17-19]. The mouse has been extensively used to study the molecular mechanisms underlying age-related sarcopenia [20]. In mammals, premature aging has been convincingly linked with ability to control inflammation *via* the thymus gland and generation of CD4+ lymphocytes [21-26]. These accumulated data show that increased thymic mass with proper programming of thymocytes contributes to a robust host immune system critical for sustained good health [12-19]. Thus, factors that stimulate thymic mass have vast biological significance and therapeutic potential in inflammation-associated health disorders.

Transcriptional factor Forkhead Box N1 [FoxN1] has been identified as a key factor in programming of a normal thymus and host immune system [27, 28]. Humans with defects in FoxN1 experience thymic atrophy and immune dysfunction, and also alopecia and mental depression. Mice absent FoxN1 expression, known as athymic nude mice, are without a functional thymus gland and consequently suffer premature aging and susceptibility to infections and cancer associated with immune dysfunction [29]. Importantly, FoxN1 therapy has been shown to stimulate thymus gland regeneration [30-32, 33] indicating potential therapeutic relevancy. Relationships between host FoxN1 expression and the microbiome have not been previously described.

*Lactobacillus reuteri* is a lactic-acid Gram-positive bacterium that colonizes the gastrointestinal tract of mammals and birds. The prototype *Lactobacillus reuteri* ATCC-PTA-6475 has been originally isolated from human's mother milk. *Lactobacillus reuteri* is considered a typical probiotic and was shown to ameliorate infectious and non-infectious gastrointestinal disorders in both humans and animals [34-36].

We have earlier shown that human *L. reuteri* inhibits cancer development in mice [37] and conveys various good health and fitness phenotypes including copious hair growth and counteraction of age-related changes in the testes and thyroid gland [38-41]. These studies had orally supplemented bacteria building upon the paradigm of the “hygiene hypothesis” such that inhabitants of developed countries have immune systems of reduced regulatory capacity due to too few microbes with refined diets, antibiotics and Caesarian births [42-44]. In this context, perinatal microbe exposures revealed transgenerational effects in offspring including a scurfy-like syndrome with athymia together with muscle wasting, scant hair growth, massive accumulations of neutrophils, and increased cancers of lungs and liver, in grandchildren animals [45]. Attempting to connect-the-dots, it was hypothesized that failure-to-thrive in progeny was due to insufficient thymogenesis and subsequent immune dysregulation predisposing to cancers later in life. Recognizing that epithelial transcription factor FoxN1 is pivotal in embryology and thymogenesis, here we tested whether microbe modulation of FoxN1 is a plausible unifying factor involving microbiota in host thrift and evolutionary success.

Here we evaluate microbial strategies to inhibit muscle wasting in murine models. We find greater muscle mass in mice consuming a beneficial microbe *L. reuteri*. We discover *L. reuteri*-associated muscle mass coincides with normalized blood neutrophil counts and retention of a youthful thymus gland size. Further, we discover FoxN1 is up-regulated in thymic epithelia after *L. reuteri* treatment commensurate with increased thymic and muscle mass. Finally, we find that nude FoxN1-deficient mice are unable to benefit from microbial treatment when compared with wild type controls. Taken together our results suggest that commensal microbiota modulate host transcriptional factors such as FoxN1 that are pivotal in mammalian fitness, survival and evolution.

## RESULTS

### Beneficial microbe *Lactobacillus reuteri* inhibits cancer-associated cachexia

**Figure 1:**
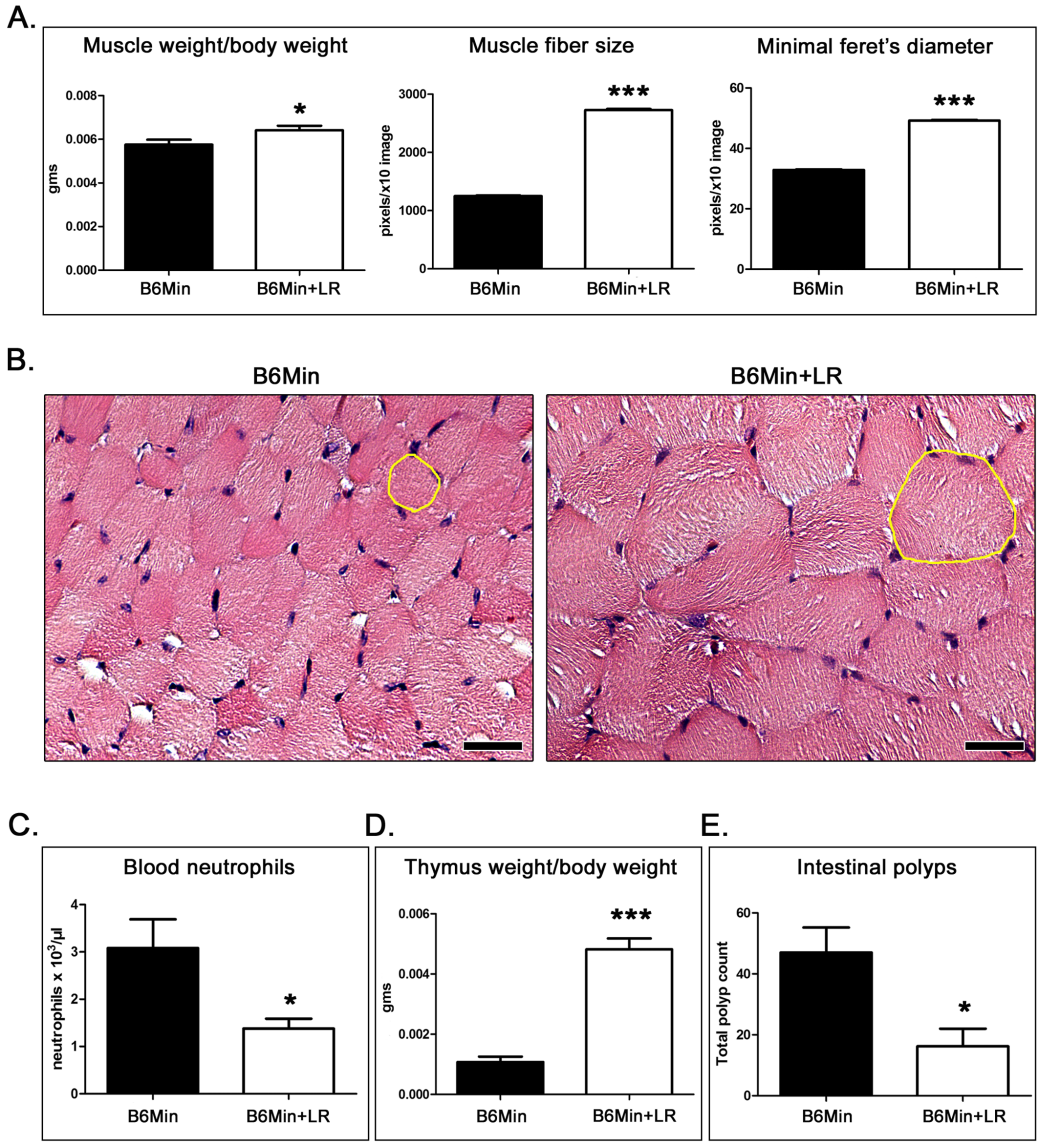
*L. reuteri* effects on gastrocnemius muscle, systemic neutrophils thymus gland and intestinal tumor burden of ApcMIN mice **A.** The muscle-to-body weight ratio and critical histomomorphometrical parameters reflecting the size of muscle fibers are significantly improved with probiotic treatment [*n* = 6]. **B.** Typical histology of gastrocnemius muscle comparing *L. reuteri*-treated mice with untreated controls [*n* = 6]. The muscle fiber cross-section profiles are larger in the treated mice. Dietary *L. reuteri* **C.** downregulates blood neutrophils **D.** rescues thymic mass and **E.** counteracts intestinal polyp formation at statistically significant levels. Numbers on the y-axis of bar graphs correspond to the mean&plusmn;SEM of the parameters assessed; *p < 0.05, ***p < 0.0001. Hematoxylin and Eosin. Scale bars: 25 μm (B)

Cancer-associated cachexia occurs in nearly half of all cancer patients, and is a leading cause of pre-mature death [1-3]. To first investigate whether individuals suffering cancer-associated cachexia may benefit from consuming beneficial microbes, we tested the widely utilized ApcMIN mouse model predisposed to cancer cachexia [46]. Twelve eight-week-old C57BL/6 ApcMIN mice were randomly subdivided into groups of six mice per treatment and then treated continuously until five-months-of-age. As expected, at the age of 5 months the ApcMIN mice we used had significantly lower body weights by comparison to their age-matched wild-type controls (ApcMIN body weight, mean&plusmn;SE = 21.48&plusmn;0.65 *vs* wild-type body weight, mean&plusmn;SE = 42.39&plusmn;2.95, *p* = 0.0014). For evaluation we selected the gastrocnemius that is a fast-twitch muscle and therefore more susceptible to cancer cachexia compared to other muscles [47]. By analyzing the gastrocnemius muscle of ApcMIN mice we found that feeding of *L. reuteri* in drinking water led to significantly (*p* < 0.05) larger gastrocnemius muscle masses (Figure 1A).

Upon histopathological examination of the gastrocnemius muscle, ApcMIN mice had overall appearance of muscle fibers with smaller cross-sectional area in untreated mice compared to the *L. reuteri*- treated ones, suggesting that *L. reuteri* protects from cancer cachexia. Microscopically, muscle fibers of untreated mice exhibited mild, occasional lesions of atrophy including vacuolated, pale and hyalinized fibers, and focal macrophage and mononuclear cell infiltration and fibrosis. These same lesions were absent from *L. reuteri*-treated mice. Likewise, internal nuclei were readily identified in muscle fibers of untreated mice; by contrast, the presence of internal nuclei was unremarkable in the mice consuming the probiotic.

To confirm and quantify this result morphometrically, we analyzed the gastrocnemius muscle fibers of the two groups of mice. We found that ApcMIN mice fed with *L. reuteri* had a significantly larger (*p* < 0.0001) mean muscle fiber cross-sectional area compared to controls (Figure 1A and 1B). We next used the minimal Feret's diameter of muscle fibers that generates a geometrical parameter that remains largely unaffected by miscalculations due to orientation and sectioning angles of muscle fibers [48]. For that, we then calculated the minimal Feret's diameter of cross-sectioned muscle fibers. Once again we found that the muscle fibers of *L. reuteri*-treated mice had a significantly larger minimal Feret's diameter compared to untreated mice (Figure 1A).

Knowing that cancer cachexia patients exhibit features of systemic inflammation [1-3], we next examined whole blood of untreated Min mice. Using circulating neutrophils as a marker for systemic inflammation, hemograms revealed that neutrophil counts were significantly elevated (*p* < 0.05) in untreated ApcMIN when compared with matched *L. reuteri*-treated mice (Figure 1C).

**Figure 2:**
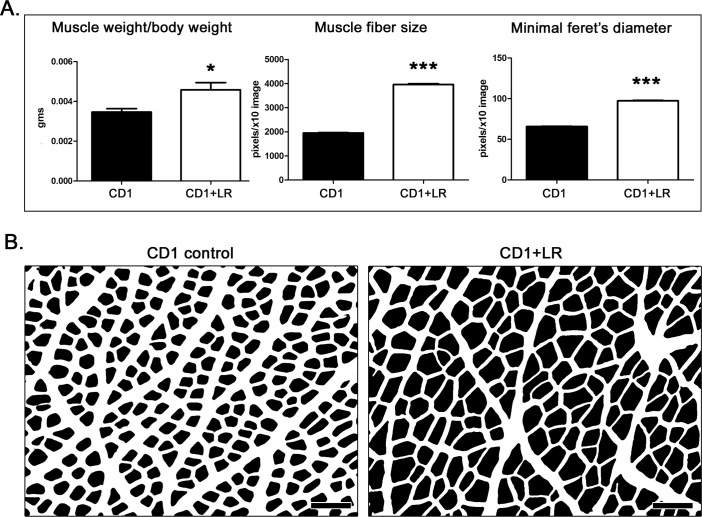
*L. reuteri* protects aged CD1 mice from senile sarcopenia **A.** The gastrocnemius weight per body weight ratio, and the means of muscle fiber area and minimal Feret's diameter are significantly higher in probiotic-treated mice [*n* = 10] compared to their age-matched controls [*n* = 10]. **B.** Final form of images used for morphometrical analysis of muscle fibers. The muscle fibers are analyzed as particles after image processing with ImageJ. Side-by-side comparison depicts larger muscle fiber profiles due to probiotic bacteria treatment. Numbers on the y-axis of bar graphs correspond to the mean&plusmn;SEM of the parameters assessed. *p < 0.05, ***p < 0.0001. Original image stain: Hematoxylin and Eosin. Scale bars: 100 μm (B)

Earlier studies have shown that ApcMIN mice exhibit uncontrollable systemic elevations in inflammatory factors IL-6, IL-1b, and TNF-a, with these same factors also associated with cachexia in patients. However, in wild type immune-competent hosts the neutrophils and other innate immune cell-related factors are productively regulated by CD4+ T lymphocytes arising in the thymus gland [44, 49-51]. Knowing that ApcMIN mice are prone to premature thymic involution [14] we next evaluated whether thymus gland size was altered in animals treated orally with *L. reuteri*. We found that the thymus gland of ApcMIN mice consuming the probiotic bacterium weighed significantly more (*p* < 0.001) compared to untreated controls (Figure 2D).

While the thymus gland produces lymphocytes that regulate host inflammatory processes and inhibit cancer, it is also understood that chronic systemic inflammation associated with cancer contributes to thymic involution [14, 52]. To address this possibility, we counted intestinal polyps in ApcMIN mice with and without feeding of probiotic. Feeding *L reuteri* was found to not only inhibit cachexia but also reduce intestinal tumor burden (*P* < 0.05) (Figure 1E). This matches earlier studies of cachexia in ApcMIN mice [7-10], and also other recent work showing that feeding of *L. reuteri* suppresses mammary, lung and liver tumor formation in mice [44, 45, 51]. This raises the chicken-and-egg question of whether it is innate immune deficits, thymic malfunction, or tumorigenesis that initiate cachexia in ApcMIN mice. It also raises the possibility that the reduced cachexia and systemic inflammation in ApcMIN mice after *L. reuteri* treatment may be due to their decreased tumor burden.

### *L. reuteri* therapy protects wild type mice from age-associated sarcopenia

In addition to diseases such as cancer, decrease of muscle mass termed ‘sarcopenia’ is one marker for the natural aging process. In order to test the protective effect of *L. reuteri* against muscle atrophy in a neoplasia-free experimental setting, we next used aged, 1-year-old CD1 wild-type mice. For these experiments, 20 outbred CD-1 mice were treated continuously starting at two-months-of-age until one-year-of-age. The gastrocnemius muscle of aged CD1 mice that were consuming *L. reuteri* weighed significantly more (*p* < 0.05) when compared with untreated control mice (Figure 2A). Likewise, the histomorphometrical analysis of this muscle showed that both the muscle fiber cross-sectional area and minimal Feret's diameter were significantly larger (*p* < 0.0001) in probiotic treated mice compared to controls (Figure 2A and 2B). This result suggests that *L. reuteri* protected the CD1 mice from senility-associated sarcopenia.

### Mice fed with *L. reuteri* have a larger thymus gland mass and increased lifespan

**Figure 3:**
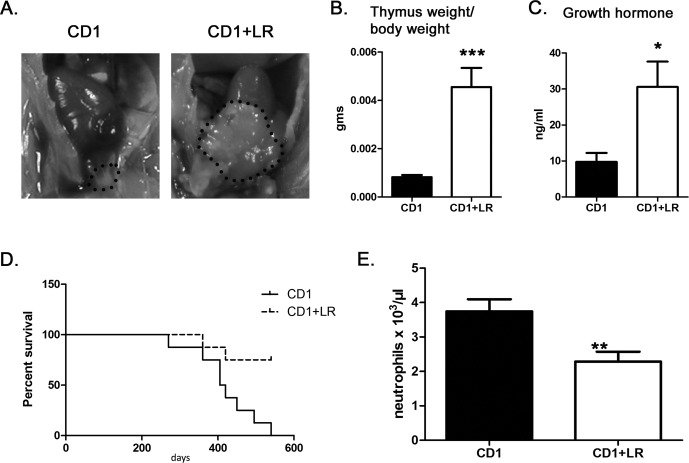
*L. reuteri* effects on thymus gland mass, longevity and systemic inflammatory tone of mice **A.** Typical gross appearance of the thymus gland in *L. reuteri*-treated [*n* = 10] and untreated control [*n* = 10] CD1 mice. Note the larger size of thymus gland in the probiotic-treated mouse. **B**. The thymus gland weight/per body weight ratio is significantly increased in CD1 mice after the consumption of the probiotic bacterium. **C**. Survival curves of CD1 mouse cohorts [n = 8 per treatment group] depicting the longevity advantage conferred by *L. reuteri* dietary supplementation. **D**. The effect of *L. reuteri* in suppressing blood neutrophils is as expected in wild-type control CD1 mice. At the same time, however, this beneficial effect is lost in FoxN1-deficient mice. The y-axis depicts the mean&plusmn;SEM of the parameters analyzed.

Building upon the finding that cachexia-prone ApcMIN mice have a larger thymus when they consume *L. reuteri*, we tested whether a similar phenomenon was also observed in the aged CD1 mice. Indeed, the thymus of probiotic-treated CD1 mice at one year of age was larger (Figure 3A) and weighed significantly more (*p* < 0.001) than the thymus of controls (Figure 3B). Knowing that thymopoiesis and cachexia have been previously linked with growth hormone [53-56], we tested serum protein levels and found significant elevations in growth hormone (Figure 3C). Increased growth hormone levels match earlier findings with other hypothalamic-pituitary hormones after oral dosing with *L reuteri* [57].

The larger muscles and thymus masses suggest that *L. reuteri* counteracts senility-associated pathologies. To access whether this had a palpable effect on the longevity of mice we then used aged CD1 mice for survival analysis. We found that mice consuming the probiotic had a statistically significant (*p* < 0.01) survival advantage compared to the untreated controls (Figure 3D). To test whether consumption of *L. reuteri* and increased longevity coincide with a decreased systemic inflammatory tone we next tested the levels of blood neutrophils of CD1 mice. We found that CD1 mice consuming the probiotic had significantly lower (*p* < 0.01) numbers of neutrophils in their blood compared to their age-matched controls (Figure 3E). This indicated that *L. reuteri* act, at least in part, by restoring host immune homeostasis and lowering systemic inflammatory tone.

### *L. reuteri* up-regulates FoxN1 expression

**Figure 4:**
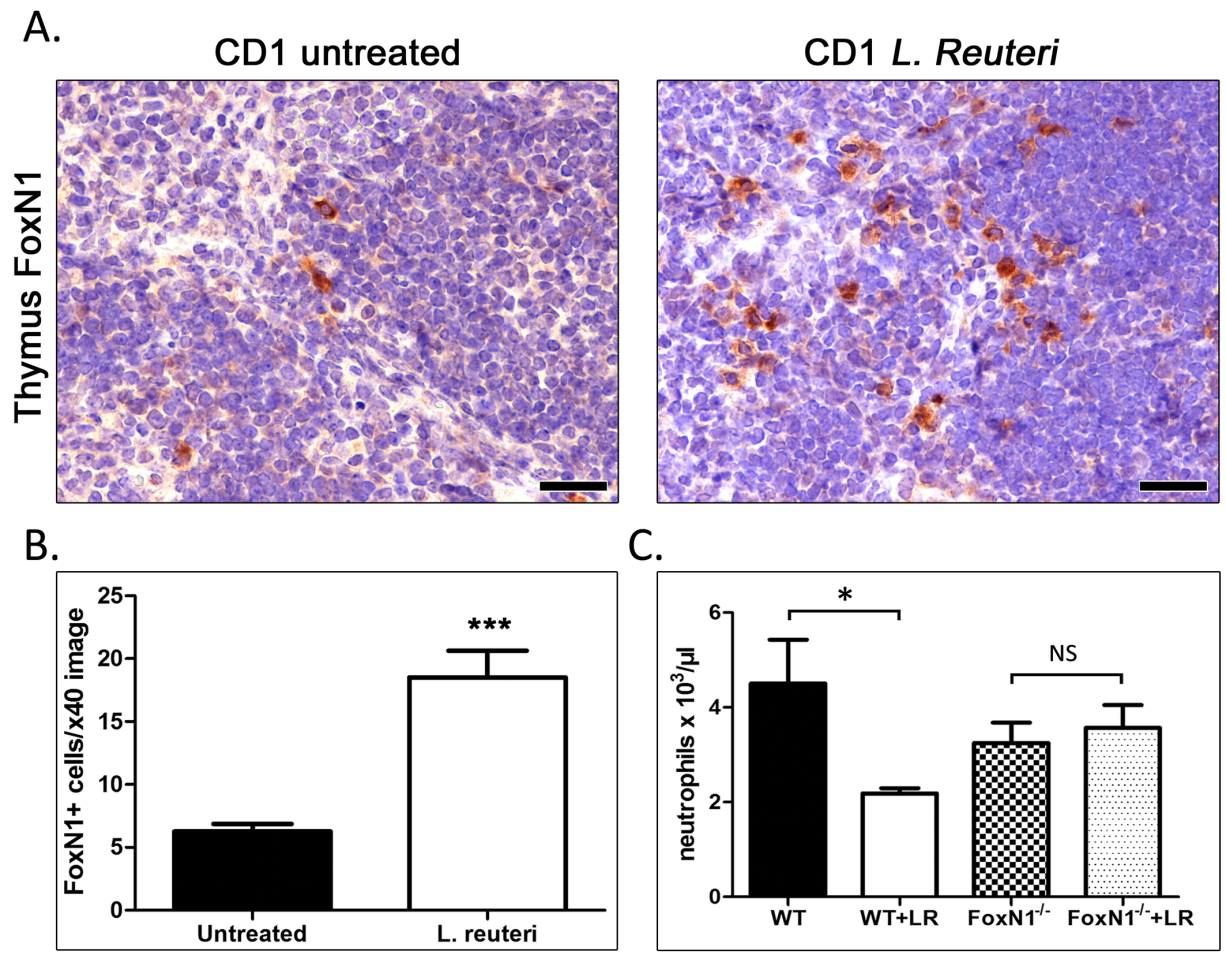
*L. reuteri* effects on thymus and systemic inflammatory tone are linked with FoxN1 **A.** FoxN1-specific immunohistochemistry on thymus gland sections of *L. reuteri*-treated [*n* = 10] and untreated mice [*n* = 10]. The FoxN-1-positive thymic epithelial cell component is denser in the probiotic-treated mice. **B**. Results of FoxN-1-positive cell morphometric counts in thymus gland. **C**. The effect of L. reuteri in suppressing blood neutrophils is as expected in control CD1 mice. This beneficial effect is lost in FoxN1-deficient mice. IHC; Diaminobenzidine chromogen, Hematoxylin counterstain. Scale bars: 25 μm (A). The y-axis of bar graphs depicts the mean&plusmn;SEM of the parameters analyzed. *p < 0.05, ***p < 0.0001.

Knowing that mice consuming L. reuteri have a larger thymus and that transcriptional factor Forkhead Box N1 [FoxN1] has been identified as key in thymic epitheliogenesis and programming of a normal thymus gland and host immune system [27, 28], we next examined FoxN1. Bredenkamp et al (2014) have recently shown shown that the inducible thymic FoxN1 expression results in robust regeneration of the involuted thymus of aged mice [32], raising the likelihood of physiological relevancy. We next, therefore, tested whether the *L. reuteri*-induced youthful thymus we observed in aged CD1 mice co-existed with elevated FoxN1 expression. For that we applied a FoxN1-specific immunohistochemical stain in thymus tissues of mice (Figure 4A). We found that the thymus of *L. reuteri*-treated mice had a significantly higher number (*p* < 0.0001) of FoxN1-positive cells by comparison with the thymus of age-matched control mice (Figure 4A and 4B). The FoxN1-positive cells were histomorphologically consistent with thymic epithelial cells and located primarily at the medulla, especially in the boundaries with the lymphocyte-rich cortex. FoxN1-positive epithelial cells, however, also existed in smaller numbers within the cortical areas.

Nude mice with a defective FoxN1 gene mimic the human condition with athymia, severe immune deficiency, and hairless skin [29]. In order to test whether microbe-induced benefits require FoxN1, we next used 3-month-old wild-type and FoxN1-deficient CD1 male mice. We found that wild type mice consuming the probiotic *L. reuteri* had significantly lower (*p* < 0.01) numbers of neutrophils in their blood compared to their age-matched controls (Figure 4C); however, nude mice absent FoxN1 showed no differences in neutrophil counts after treatment with *L. reuteri* (Figure 4C).

### FoxN1 is required for gut microbe-associated retention of muscle mass

**Figure 5:**
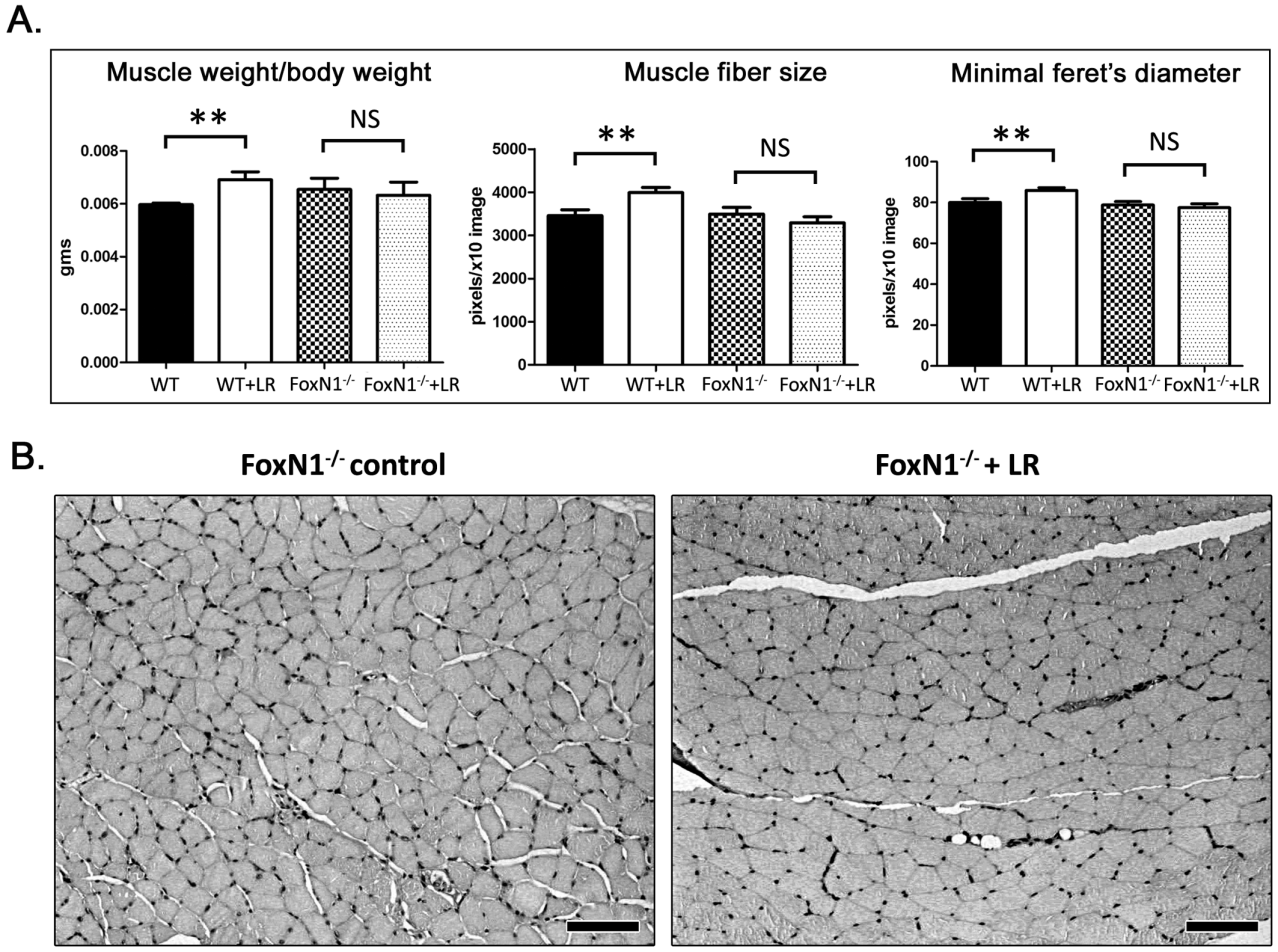
The beneficial effect of *L. reuteri* on gastrocnemius muscle depends upon intact FoxN1 gene **A.** While wild-type control mice [*n* = 5] present the standard improvements in the muscle-to-body weight ratio and muscle fiber size due to the probiotic treatment [*n* = 5], FoxN1-deficient mice [*n* = 5 per treatment group] fail to replicate the same phenomenon. **B.** The gastrocnemius muscle fibers of both *L. reuteri*-treated and untreated FoxN1-deficient mice have a comparable size. Numbers on the y-axis of bar graphs correspond to the mean&plusmn;SEM of the parameters assessed. **p < 0.001, NS: *p* > 0.05. Original image stain: Hematoxylin and Eosin. Scale bars: 100 μm. To increase the visibility of muscle fiber boundaries the blue channel of the original HE image was obtained using the H&E color deconvolution plugin of ImageJ. The image product was then transformed in grayscale and uniformly enhanced using the levels command in Photoshop (B)

Having shown that the ability of edible *L. reuteri* in downregulating systemic neutrophils depends upon intact *FoxN1*, we next used the same mouse model to test whether FoxN1 is also needed for the beneficial effects of the probiotics on skeletal muscles. For these studies, ten athymic nude [CD-1 genetic background] mice and ten age-matched CD-1 controls were randomly subdivided [*n* = 5 mice per group] and then treated continuously starting at eight-weeks-of-age for a duration of four weeks. The gastrocnemius muscle of *L reuteri*-treated wild-type mice was significantly (*p* < 0.01) heavier compared to untreated controls (Figure 5A). Thus, dietary supplementation with this microbe resulted in larger muscles in wild-type mice. At the same time, however, the LR effect on gastrocnemius weight was negated in recipient mice lacking the *FoxN1* gene (Figure 5A). To elaborate on this result we analyzed morphometrically the gastrocnemius muscle fibers. In wild-type mice the LR treatment led to a statistically significant increase of both muscle fiber size (*p* < 0.01) and Feret's diameter (*p* < 0.01). By contrast, the same morphmetrical parameters were comparable between LR-treated and non-treated FoxN1-deficient mice (Figure 5A and 5B).

## DISCUSSION

**Figure 6:**
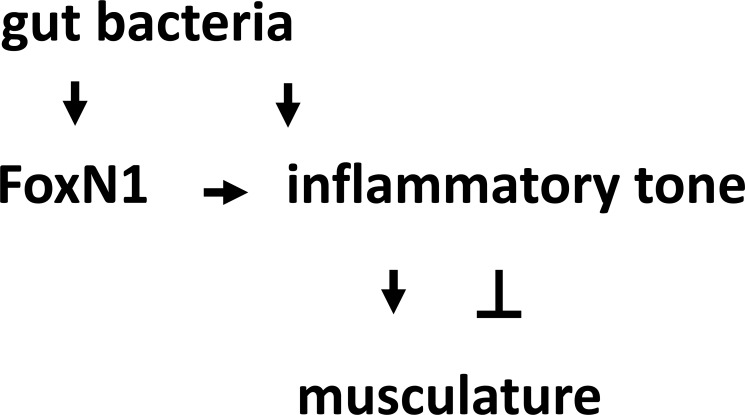
Conceptual overview Routine oral dosing of microbes inhibits muscle-wasting syndromes including cachexia and sarcopenia. Microbiota stimulate thymopoiesis and regulate systemic inflammatory tone together with muscularity by a FoxN1-dependent mechanism.

Here we evaluate microbial strategies to inhibit muscle wasting in the context of cancer cachexia and also during normal aging. We discover in both model systems an increased muscle mass in mice consuming a beneficial human commensal microbe *Lactobacillus reuteri*. Along with lower risk for muscle wasting, routine feeding of *L. reuteri* in drinking water reduces blood neutrophil counts used as a surrogate marker for systemic inflammation. At the same time, mice eating *L. reuteri* also exhibit increased thymus gland size commensurate with up-regulation of Forkhead Box [Fox]N1 in thymic epithelia after *L. reuteri* therapy. Finally, in order to probe the molecular mechanism, we find that FoxN1-deficient athymic nude mice were unable to benefit from microbial treatment. Taken together our results suggest that commensal microbiota modulate host transcriptional factors such as FoxN1 that are pivotal in host fitness and survival by regulating host inflammatory tone to impart balanced immune responses (Figure 6). In this way, microbiota offer a tractable target to promote systemic homeostasis lowering risk of muscle wasting and other inflammation-associated morbidity.

A key finding is that dietary supplement with a microbe, *L reuteri*, prevented muscle atrophy in mice. In future studies it will be interesting to examine body weight over time and functional criteria such as grip strength for improvement after intake of bacteria. In earlier studies it was shown that general food consumption was unaltered by feeding *L reuteri* to mice [39], but that was not specifically examined here. While in the present study *L. reuteri* prevented senility-associated muscle atrophy in aged CD1 mice, muscle cachexia in the ApcMIN mouse model is more complicated. *L. reuteri* treatment in ApcMIN mice reduced the intestinal tumor burden similarly to what has been previously observed in other types of tumors [37, 45]. This makes it difficult to assess the extent of the reduced tumor burden contribution in the prevention of cancer-associated cachexia. Further studies using *L. reuteri* treatment in different mouse models of cachexia are needed to shed light on this probiotic effect.

Cachexia has been associated with chronic systemic inflammation, in particular involving neutrophils, in health maladies such as cancer or COPD [4]. Blood neutrophil counts are one widely used surrogate marker for systemic inflammation. Several lines of evidence implicate GI tract microbiota stimulating neutrophils and other cells of innate immune cells leading to tumor formation throughout the body [11, 49, 58-64]. In order to elucidate systemic immune balance that inhibits such diseases, prior studies have focused upon reciprocal systemic relationships between neutrophils and T lymphocytes of adaptive immunity [37, 60, 65-70]. In the context of cancer, neutrophils have been identified in animal models and humans as an important factor in cancer initiation and development [60, 67, 71-79]. To restore and maintain good health, innate immune networks [such as those involving neutrophils] are persistently regulated by anti-inflammatory activities of CD4+ T_REG_ [49, 59, 80]. According to this line of reasoning, more frequent inflammation-associated cancers arise with a weakened T_REG_-mediated inhibitory loop with consequences of neutrophilia and elevated IL-6 heightening the risk of cachexia [51]. In prior studies, microbial phenotypes were transplantable using highly purified CD4+ lymphocyte subsets [49, 58, 59, 81]. It remains to be determined which specific T lymphocyte subsets participate in muscle-related homeostatic processes.

During youth, the site of T lymphocyte maturation is the thymus, and the thymus gland also serves to routinely balance the activities of a normally functioning immune system during adulthood. Observation of enlarged thymus glands after eating *L. reuteri* in the present study wasn't entirely surprising. After all, the present focus upon the thymus emerged from earlier ‘hygiene hypothesis’-based studies, revealing repairable transgenerational phenomena of a scurfy-like athymia implicating perinatal microbial effects on the infant thymus [45]. In mammals, premature thymic involution has been convincingly linked with a wide spectrum of immune disorders resulting in failure to distinguish self from non-self [22, 23] and inability to counteract infectious diseases and cancer. Similar spontaneous thymic atrophy has been described in ApcMIN mice [14]. Thymic atrophy dovetails with observations of the ‘hygiene hypothesis’, such that inhabitants of developed countries have immune systems with reduced lifelong immune regulatory capacity coinciding with antibiotics and Caesarian births [42-44]. A common denominator is that impaired immune regulatory capacity leads to uncontrolled inflammatory responses and ultimately inflammation-associated cancers and other disabilities later in life [43-45]. The current observations involving cachexia match these earlier studies using dietary enrichment with beneficial bacteria [37, 39]. It's interesting the extent to which hypothalamic-pituitary hormone levels, such as growth hormone in the present study, appear to overlap in thymic health and homeostasis [53]. Oxytocin [57, 82, 83], testosterone [40], and thyroid hormone [41] were also previously found to be modulated by ingested bacteria may impact host muscle mass in these studies. Oxytocin may directly or indirectly help sustain musculature [57, 82, 83].

One important new finding is the increase in FoxN1 protein expression in thymic tissue after consuming probiotic bacteria. FoxN1 is part of the forkhead family or winged-helix transcription factors [84] that helps control production of T lymphocytes. *FoxN1* is a master regulator in the thymic epithelial cell [TEC] lineage specification in that it promotes transcription of down-stream genes, which, in turn, regulate TEC differentiation. In particular, *FoxN1* mainly regulates TEC patterning in the fetal stage and TEC homeostasis in the post-natal thymus [28]. Even during normal aging there is gradual decline of the thymus and immune system resulting in insufficient adaptive immunity predisposing to infections and cancer. In this way, the thymus gland has been proposed as a ‘Fountain of Youth’ for its key roles in immune system and consequently systemic health. By artificially stimulating *FoxN1*, it's been possible to rebuild the senescent thymus of aged mice[31, 32]. This raises that likelihood that targeting microbiota to modulate Wnt signaling and elevate FoxN1 will help sustain youthful thymic activities.

The *FoxN1* gene and protein are also directly or indirectly important in many other developmental processes, immune system regulation, metabolism, cancer and aging [84]. Embryologically, the FoxN1-rich tissue originates from the epithelium of the third pharyngeal pouch [85]. The encoded protein is proposed to also regulate differentiation of keratinocytes giving athymic nude mutant mice their hairless appearance. It is noteworthy that luxuriant fur - a feature of FoxN1 protein up-regulation - is also a prominent phenotype in mice eating *L. reuteri* [38]. As a result of the immune and follicular [hair production] defects, FoxN1-deficient nude mice are widely used as a model system in oncology, immunology, dermatology, and transplantation studies. Female nude mice also have underdeveloped mammary tissue and are ineffective at nursing their young [86]. In humans, *FoxN1* expression is high in cerebral tissue, and brain alterations have been described in fetuses carrying the *FoxN1* homozygous mutation [87]. In particular, *FoxN1* is highly expressed in glial cells that make important contributions in human intelligence [88]. FoxN1 is linked with the Wnt signaling pathway providing one possible ontological and systemic mechanism linking microbes with thymogenesis [89]. Systemic growth hormone levels have also been implicated in muscle development and thymogenesis [53].

Recognizing that epithelial transcription factor FoxN1 is pivotal in mammalian thymus, hair follicles, and mammary gland development, we reasoned that environmental microbe modulation of FoxN1 is a plausible unifier of microbiota in ontogeny and phylogeny [90]. *FoxN1* expression in thyroid gland tissue may help explain up-regulation of blood T4 after consumption of *L. reuteri* by mice [41]. In addition to thyroid function, FoxN1 has several key common denominators with mammalian evolution, survival and fitness: a) thymogenesis with exquisite control of self-*vs*-nonself in sustained placental pregnancy, b) robust pellage for thermal regulation, and c) mammogenesis for nutrition for placental offspring. Together these support the notion that microbe-host interactions involving FoxN1 provide the pillars for mammalian life.

In conclusion, microbiota may offer novel strategies to reduce muscle wasting and restore good systemic health. Our discovery that edible bacteria have beneficial effects on the muscle mass of mice provides elementary evidence for the presence of a gut microbiota-skeletal muscle axis in mammals. Further research of the mechanisms behind this novel axis and tests for its existence in humans may offer new alternatives for prevention of cancer-associated cachexia and other muscle wasting disorders. Commensurate up-regulation of FoxN1 in thymic epithelia by oral microbe therapy unifies bacteria and immune homeostasis. We have previously shown that sterile microbe preparations are sufficient to inhibit carcinogenesis in mice [44], and therapeutic potential of microbial fractions remains to be examined in the setting of cachexia. Taken together our results suggest that commensal microbiota modulate host resiliency *via* FoxN1 to regulate host inflammatory tone for effective, yet balanced, immune responses with rapid restoration of homeostasis afterwards. In this way, microbiota offer a tractable target to impart systemic homeostasis lowering risk of inflammation-associated morbidity.

## MATERIALS AND METHODS

### Animals

All animals were housed in Association for the Assessment and Accreditation of Laboratory Animal Care (AAALAC)- approved facilities and maintained with approval by the Institutional Animal Care and Use Committee (IACUC) at Massachusetts Institute of Technology. *Apc^Min/+^* mice [ApcMIN] on a C57BL/6J background were originally obtained from the Jackson labs and bred in-house to provide *Apc^Min/+^* and *wildtype (wt)* littermates for experiments involving cancer cachexia. Outbred conventional CD-1 Swiss stock mice (Charles River; Wilmington, MA) were utilized for aging studies absent any transgenic predilections to cancer. In order to test the relevancy of FoxN1, athymic homozygous nude mice Crl:NU(NCr)-*Foxn1^nu^* (Charles River; Wilmington, MA) were challenged with oral microbe administration starting at the age of eight weeks. Each experiment included 5-10 animals per sex per treatment group, performed in duplicate, as described in detail below.

### *L. reuteri* administration

In each experiment subsets of mice received in their drinking water a strain of *Lactobacillus reuteri* ATCC-PTA-6475 cultivated as described elsewhere [57, 91]. Live organisms were supplied at a starting dosage of 3.5×10^5^ organisms/mouse/day in drinking water with live bacterial counts in water bottles calculated as described in detail in Lakritz et al (2014) [37]. Control mice received regular drinking water. Fresh drinking water for both groups of animals was replaced twice weekly throughout the experiments.

### Experimental design

#### Experiment 1

To probe the roles of microbiota in cancer cachexia, twelve eight-week-old C57BL/6 *Apc^Min/+^* [ApcMIN] mice were randomly subdivided into groups of six mice per treatment and received in their drinking water *Lactobacillus reuteri* ATCC-PTA-6475 as described above and elsewhere [57, 91] continuously until five-months-of-age.

#### Experiment 2

To test whether oral therapy with gut microbes impacts muscle wasting during normal aging and independent of cancer, we next examined 20 outbred CD-1 mice. Experimental mice received in their drinking water *L reuteri* ATCC-PTA-6475 as described above starting at two-months-of-age until one-year-of-age.

#### Experiment 3

To test whether FoxN1 is required for muscle-building benefits of oral therapy with gut microbes, we next examined ten athymic nude CD-1 mice and ten age-matched CD-1 controls. Experimental mice were randomly subdivided and then received in their drinking water *L reuteri* ATCC-PTA-6475 as described above starting at eight-weeks-of-age for a duration of four weeks.

### Necropsy and sample collection

Mice underwent necropsy after CO_2_ overdose and exsanguination. Whole blood, the gastrocnemius muscle and the thymus gland were collected for various analyses. Intestinal samples were flattened for macroscopic counts of intestinal polyps.

### Complete blood cell counts

Whole blood was collected by cardiac puncture from unconscious animals upon necropsy and suspended in EDTA to prevent clotting. Automated neutrophil counts were then performed using mouse parameters in a HemaVet 950FS (Drew Scientific, Oxford CT).

### Serum growth hormone levels

Whole blood was collected by cardiac puncture from unconscious animals upon necropsy to derive serum for hormone assays. Serum growth hormone levels in aged CD-1 mice were tested by radioimmunoassay (AniLytics Corp, Gaithersburg MD).

### Determining mass of skeletal muscle and thymus gland

Upon necropsy, the entire mouse, plus their left gastrocnemius muscle and whole thymus gland were weighed using a ScoutPro SP202 scale [Chaus Corporation, Pinebrook NJ].

### Histopathology and immunohistochemistry

For histologic evaluation, formalin-fixed gastrocnemius muscles and thymus glands were embedded in paraffin, cut at 4 μm, and stained with hematoxylin and eosin or immunohistochemistry. For morphometry, cross sections from the midline of the gastrocnemius muscle of six randomly selected mice per experimental group were used. Two x10 magnification images were captured from standardized areas of each gastrocnemius cross section. The muscle fiber area and Feret's diameter were automatically calculated in each image using the “analyze particles” command of the ImageJ image processing and analysis program (NIH, Bethesda, MD) based on a previously described methodology. A total of approximately 4000 muscle fibers were analyzed per experimental group. FoxN1-specific immunohistochemistry was performed with rabbit anti-FoxN1 polyclonal antibodies (antibodies-online GmbH, Aachen, Germany). Heat-induced antigen retrieval was performed with EDTA buffer, pH 8. The IHC stain and quantitative histomorphometry of IHC-positive cells in x40 high magnification images was done as previously described [57].

### Statistical analyses

Data were compared between groups using Mann-Whitney U analysis. Statistical significance was set at *P* < 0.05. All analyses were performed with the Graphpad Prism version 5.0 for windows, GraphPad software, San Diego
