# Correction: Beneficial bacteria inhibit cachexia

**DOI:** 10.18632/oncotarget.25750

**Published:** 2018-06-29

**Authors:** Bernard J. Varian, Sravya Gourishetti, Theofilos Poutahidis, Jessica R. Lakritz, Tatiana Levkovich, Caitlin Kwok, Konstantinos Teliousis, Yassin M. Ibrahim, Sheyla Mirabal, Susan E. Erdman

**Affiliations:** ^1^ Division of Comparative Medicine, Massachusetts Institute of Technology, Cambridge, MA, USA; ^2^ Laboratory of Pathology, Faculty of Veterinary Medicine, Aristotle University of Thessaloniki, Thessaloniki, Greece

**This article has been corrected:** The corrected author name is given below:

**Sravya Gourishetti**

Original article: Oncotarget. 2016; 7:11803-11816. https://doi.org/10.18632/oncotarget.7730

